# Advances in live-imaging of mouse development: exploring the researcher’s interdisciplinary tool-kit

**DOI:** 10.1242/dev.199433

**Published:** 2021-09-16

**Authors:** Matthew J. Stower, Shankar Srinivas

**Affiliations:** 1 Department of Physiology, Anatomy and Genetics, University of Oxford, South Parks Road, Oxford OX1 3QX, UK

## Abstract

Live-imaging of embryogenesis is an important part of the developmental biologist’s armoury of methods. In this article, we focus on the use of live-imaging specifically in understanding early mouse embryogenesis. Advances in several disciplines including embryo culture, microscopy hardware and computational analysis have all contributed to our ability to probe dynamic events during early mouse development. Together, these comprise a versatile and powerful ‘tool-kit’, enabling us to not only image events during embryogenesis, but also intervene in them. Coordinating advances on all these fronts will greatly facilitate the use of live-imaging for investigating mouse embryogenesis.

## Introduction

**Fig 1:**
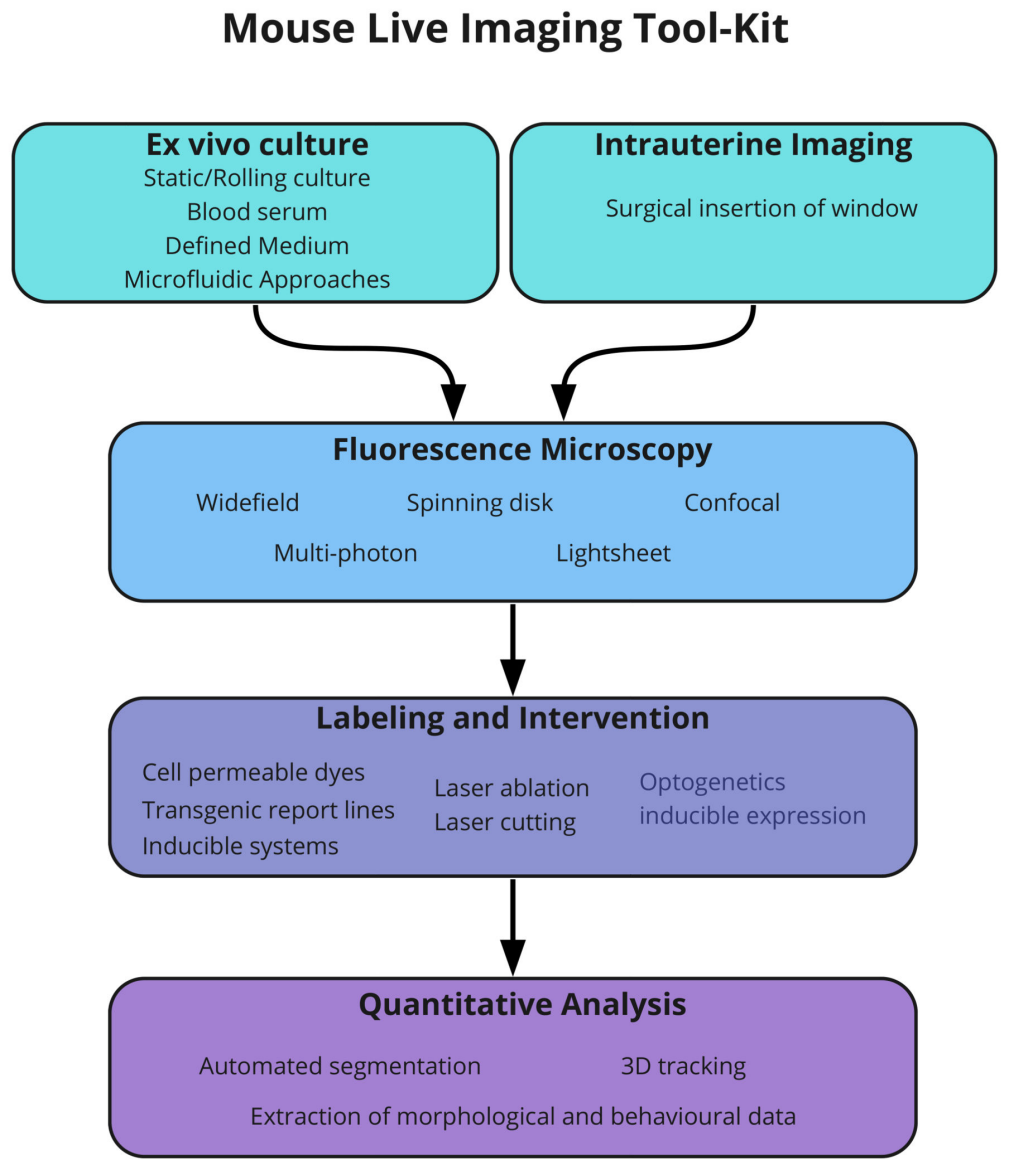
The researcher’s ‘tool-kit’ with regard to live-imaging. Advances on all these fronts will greatly facilitate the use of live-imaging for investigating mouse embryogenesis.

The first live imaging “cinematographs”, using film to record mouse development through a microscope, were of pre-implantation embryos undergoing cleavage as far as the blastocyst stage (Kuhl and Friedrich-Freska 1936). Due to the difficulty in developing consistent *in vitro* culture conditions that supported mouse development, it wasn’t until the 1960’s that detailed time-lapse films of early mouse embryonic development were achieved by multiple labs (Borghese and Cassini 1963; Cole 1967; Mulnard 1967). Since these pioneering imaging experiments, huge strides in a wide range of technologies have revolutionized our ability to image live, developmental processes in the mouse embryo. Our ability to gain insights into mammalian development has been facilitated by parallel advances in: culture technology; microscopy hardware; cell and tissue labelling approaches combined with the ability to optically intervene in a controlled manner and; computational approaches to analyse image data. Here, we explore key aspects of the researcher’s ‘tool-kit’ (Fig. 1) with regard to imaging early mouse embryogenesis. We highlight how live-imaging is an intrinsically interdisciplinary endeavour and the importance of advancing each of these complimentary technologies, any one of which can become limiting. This is not a comprehensive review of live imaging technologies, for which we point the reader to several reviews in this special issue and the existing literature. We apologise in advance to colleagues whose contributions we were unable to reference in this brief spotlight article.

### Mouse Embryo Culture for Live imaging

Due to the intrauterine development of mammalian embryos, a fundamental part of the live-imaging tool-kit, as it relates to the mouse, are approaches to culture embryos *ex utero*. Isolation and *in vitro* culture of mouse pre-implantation embryos is relatively easy and has become widely used, with embryos cultured from the 1-cell to blastocyst stage using static culture conditions and defined media, enabling live-imaging on a range of microscopic set-ups (Hiiragi and Solter 2004; McDole and Zheng 2012; Strnad et al. 2016; Watanabe et al. 2014). At post-implantation stages, well established approaches exist for maintaining embryos using roller culture, where the continuous rolling is understood to promote gas exchange, but precludes contemporaneous imaging (Beddington 1987; Lawson et al. 1986; Tam 1998). Static culture approaches were therefore developed to image post-implantation embryos, for example to study AVE cell migration (Srinivas et al. 2004), and to follow cell movements over several days continuously during gastrulation (McDole et al. 2018). Recently, such approaches have been extended to culture mouse embryos from pre-gastrulation stages for 5-6 days using a combination of static and roller culture (Aguilera-Castrejon et al. 2021). While this set-up does not currently incorporate continuous long-term live imaging capability, it take us one step closer to developing other approaches, for example with microfluidic chip based devices, that have been used in cell culture experiments (Coluccio et al. 2019) to give precise control over culture conditions, thereby potentially enabling long-term culture along with live imaging.

### Advances in Microscopy hardware

Microscopy hardware is naturally a key component of the live imaging tool-kit. Live imaging of mouse embryos during development is a delicate balancing-act; using a limited photon budget to achieve sufficient signal-to-noise and maximise spatial/temporal resolution, while minimising photo-toxicity so as to ensure normal development. The application of fluorescent microscopy to developmental biology, using electromagnetic radiation to excite fluorophores labelling a specific structure or cell-type, has enabled dynamic cellular and sub-cellular processes to be visualised, revolutionising our understanding. However, exposure of embryos to the illumination required to excite fluorescence can lead to photothermal and photochemical damage, arising from the heating of the sample and the generation of free radicals respectively. For example, while single-photon confocal microscopy has the advantage of optically sectioning a sample, it still exposes the entire depth of the sample to illumination. This limits its use for live imaging of early mouse development. Multi-photon microscopy reduces phototoxicity by using near-infrared wavelength pulsed laser excitation, (Benninger and Piston 2013). Here, a fluorophore is only excited when two low energy photons are absorbed. This non-linear effect only occurs at the focal point, thereby providing optical sectioning while also restricting photochemical damage. Such multi-photon microscopy has been used to good effect for studying sensitive pre-implantation stages at high resolution (McDole and Zheng 2012).

An increasingly popular way of minimising photodamage is using lightsheet microscopy. This technique was first described using a brightfield illumination with a slit-aperture in the early 1900’s (Siedentopf and Zsigmondy 1903), and adapted to fluorescence microscopy by using a cylindrical lens or scanning laser beam to create a thin “light-sheet” for illuminating the sample (Huisken et al. 2004; Keller and Stelzer 2008). By positioning the excitation lightsheet orthogonal to the imaging objective and placing the sample in this sheet, the embryo can be imaged, with only the plane being imaged being illuminated. Optically sectioning through the entire sample requires moving the sample through the fixed sheet of light that is positioned at the focus of the detection objective. This set-up enables full image volumes to be rapidly acquired while minimising photodamage, allowing higher temporal resolution and longer duration than generally possible with confocal or multi-photon microscopy. Additionally, multiple views of the embryo can be captured by rotating the sample, or alternately, in instruments with two imaging objectives, from two angles acquired simultaneously (Huisken and Stainier 2007; Krzic et al. 2012). Such multi-view imaging therefore enables one to image further into the sample, as the highest quality images from each view angle can subsequently be computationally combined into a single volume. Lightsheet imaging has been used to generate high-resolution 3D whole-volume images of mouse embryos and has been used to image pre-implantation development (Strnad et al. 2016), early gastrulation (Ichikawa et al. 2013; Mathiah et al. 2020), and into post-gastrulation stages (McDole et al. 2018; Udan et al. 2014). Lightsheet microscopy has been a little slow to be adopted for mouse development due to the challenging aspects of suspending the sample between the objectives. Several commercial systems are now available with an inverted set-up that allow one to image the sample in a dish, for example InVi SPIM (Bruker-Luxendo) and the Lattice Lightsheet 7 (Zeiss). Furthermore, modifications incorporating multi-photon (Truong et al. 2011), beam-shaping (Chen et al. 2014) and airy scanning technology (Vettenburg et al. 2014) have also been developed. These next-generation lightsheet microscopes, integrated with advanced culture methodologies, have the potential to become an established part of mouse embryologists’ tool-kit in the future.

### Genetic labelling and optical modulation of cell function

In combination with advances in microscopy hardware, the ability to label cells with genetically encoded fluorescent proteins has revolutionised the field of live imaging. The use of genetically modified mouse fluorescent reporter lines can provide temporal and spatial information of gene expression, cell lineage, and the position and behaviour of labelled proteins (see (Xenopoulos et al. 2012) for review). Furthermore, mouse lines exist that encode sensors of specific behaviour (e.g., cell cycle progression (Abe et al. 2013). To achieve mechanistic understanding of mouse development, some sort of perturbation or intervention is required. In the context of live-imaging, this has generally been achieved through live imaging of genetic knockout mutant embryos, or through the exposure of embryos to pharmacological inhibitors. However, there can be drawbacks to these approaches; inhibitors may affect all cells in the embryo making it difficult to disentangle their effect on different cells, or tissues and knock-outs of genes in mouse embryos, even if generated in a tissue-specific manner, may have such a large effect that it becomes difficult to interpret the phenotype. One approach that can provide more precise spatiotemporal control, enabling localised acute intervention, is laser-ablation. This allows one to not only ablate entire cells but also break sub-cellular structures such as actin-myosin cables, to provide insight into mechanical forces within tissues. This has been widely used in drosophila (Kiehart et al. 2000; Shivakumar and Lenne 2016) and also been adapted for mouse embryos (Angelo and Tremblay 2013; Fierro-Gonzalez et al. 2013). Going forward optogenetic approaches to enable genes to be switched on and off in groups of cells (Konermann et al. 2013), or to control the localisation of proteins within cells (Buckley et al. 2016) combined with live imaging technology, have the potential to transform the precision with which we can intervene in cellular processes during mouse development.

### Quantitative analysis of image data

Advances in embryo culture, microscopy hardware and labelling technology allow us to record developmental processes in great detail. However, one increasingly frequent limiting factor in fully exploiting these technologies is in our ability to computationally analyse the large and complex multi-dimensional datasets that can be generated. For example, multi-view lightsheet imaging experiments of mouse embryos developing over several days can generate 10 terabytes *per embryo* (McDole et al. 2018). Such datasets require dedicated computational approaches for both image processing (fusing image from multiple view angles, spatiotemporal registration, image augmentation) and data-analysis (cell tracking in 3D-space of thousands of cells, across hundreds of data-points). Machine learning based approaches to automate analysis are proving effective and several software packages have been developed for such image processing and 3D cell-tracking requirements, including MaMut (Wolff et al. 2018), BigDataViewer (Pietzsch et al. 2015) and RACE (Stegmaier et al. 2016).

**Fig 2:**
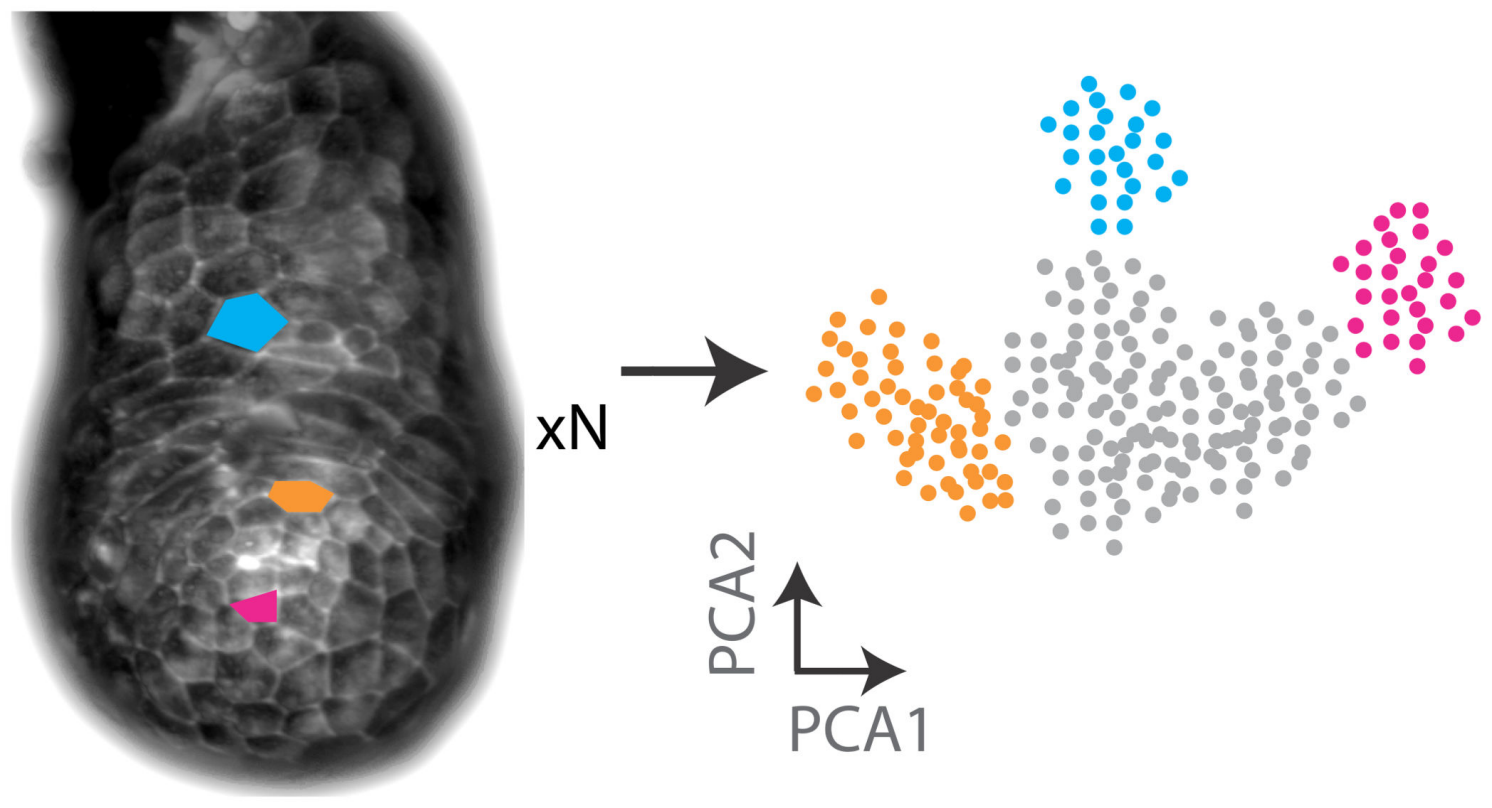
Quantitative analysis of cell behaviour from multiple embryos requires new ways of considering and displaying complex multi-dimensional image data. In this illustration, three cells with different geometric and behavioural properties have been coloured in the lightsheet image of a pre-gastrulation embryo at left, and cells with quantitatively similar characteristics to them are depicted in the plot at right.

It is clear that a major challenge for future live imaging-based studies will be the development of bespoke computational analysis pipelines to extract and analyse specific behaviours. For example, a quantitative understanding of tissue remodelling at the single-cell level will require approaches for integrating high-resolution image data from multiple embryos within an analytical framework that can accommodate natural variations in mouse embryo size, morphology, and developmental timing. It will also require a way to consider and display such complex data in a unified space, for which we can take inspiration from the approaches used to analyse multi-dimensional data in single cell ‘omics studies (Fig 2).

### Conclusion

Live imaging of mouse development is on the cusp of a “golden-age” due to a convergence of several factors; new microscopy technologies, mature genetic reporter technologies, and novel experimental set-ups that provide researchers with exquisite spatial and temporal control over experimental interventions. These are being “turbo-charged” by the development of novel analytical approaches to extract quantitative insights into cell behaviour during development. Ultimately, the coordinated development of these complimentary technologies will start to the push the field into the realms of ‘Big-Data’ science. Exciting times indeed!

## Summary Statement

Live-imaging is an intrinsically interdisciplinary endeavour, dependent on advances in several disciplines including embryo culture, microscopy hardware and computational analysis, any one of which can become limiting.

## References

[R1] Abe T (2013). Visualization of cell cycle in mouse embryos with Fucci2 reporter directed by Rosa26 promoter. Development.

[R2] Aguilera-Castrejon A (2021). Ex utero mouse embryogenesis from pre-gastrulation to late organogenesis. Nature.

[R3] Angelo JR, Tremblay KD (2013). Laser-mediated cell ablation during post-implantation mouse development. Dev Dyn.

[R4] Beddington R, Monk M (1987). Mammalian Development: A Practical Approach.

[R5] Benninger RK, Piston DW (2013). Two-photon excitation microscopy for the study of living cells and tissues. Curr Protoc Cell Biol.

[R6] Borghese E, Cassini A, Rose CG (1963). Cinematography in Cell Biology.

[R7] Buckley CE (2016). Reversible Optogenetic Control of Subcellular Protein Localization in a Live Vertebrate Embryo. Dev Cell.

[R8] Chen BC (2014). Lattice light-sheet microscopy: imaging molecules to embryos at high spatiotemporal resolution. Science.

[R9] Cole RJ (1967). Cinemicrographic observations on the trophoblast and zona pellucida of the mouse blastocyst. J Embryol Exp Morphol.

[R10] Coluccio ML (2019). Microfluidic platforms for cell cultures and investigations. Microelectronic Engineering.

[R11] Fierro-Gonzalez JC (2013). Cadherin-dependent filopodia control preimplantation embryo compaction. Nat Cell Biol.

[R12] Hiiragi T, Solter D (2004). First cleavage plane of the mouse egg is not predetermined but defined by the topology of the two apposing pronuclei. Nature.

[R13] Huisken J, Stainier DY (2007). Even fluorescence excitation by multidirectional selective plane illumination microscopy (mSPIM). Opt Lett.

[R14] Huisken J (2004). Optical sectioning deep inside live embryos by selective plane illumination microscopy. Science.

[R15] Ichikawa T (2013). Live imaging of whole mouse embryos during gastrulation: migration analyses of epiblast and mesodermal cells. PLoS One.

[R16] Keller PJ, Stelzer EH (2008). Quantitative in vivo imaging of entire embryos with Digital Scanned Laser Light Sheet Fluorescence Microscopy. Curr Opin Neurobiol.

[R17] Kiehart DP (2000). Multiple forces contribute to cell sheet morphogenesis for dorsal closure in Drosophila. J Cell Biol.

[R18] Konermann S (2013). Optical control of mammalian endogenous transcription and epigenetic states. Nature.

[R19] Krzic U (2012). Multiview light-sheet microscope for rapid in toto imaging. Nat Methods.

[R20] Kuhl W, Friedrich-Freska H (1936). Richtungskörperbildung und Furchung des Eies sowie das Verhalten des Trophoblasten de weißen Maus. Zool Ans Suppl.

[R21] Lawson KA, Meneses JJ, Pedersen RA (1986). Cell fate and cell lineage in the endoderm of the presomite mouse embryo, studied with an intracellular tracer. Dev Biol.

[R22] Mathiah N (2020). Asymmetry in the frequency and position of mitosis in the mouse embryo epiblast at gastrulation. EMBO Rep.

[R23] McDole K, Zheng Y (2012). Generation and live imaging of an endogenous Cdx2 reporter mouse line. Genesis.

[R24] McDole K (2018). In Toto Imaging and Reconstruction of Post-Implantation Mouse Development at the Single-Cell Level. Cell.

[R25] Mulnard JG (1967). [Microcinematographic analysis of the mouse egg development from stage 2 to the blastocyst]. Arch Biol (Liege).

[R26] Pietzsch T (2015). BigDataViewer: visualization and processing for large image data sets. Nat Methods.

[R27] Shivakumar PC, Lenne PF (2016). Laser Ablation to Probe the Epithelial Mechanics in Drosophila. Methods Mol Biol.

[R28] Siedentopf H, Zsigmondy R (1903). Uber sichtbarmachung und Größenbestimmung ultramikoskopischer teilchen, mit besonderer anwendung auf goldrubingläser. Annalen der Physik.

[R29] Srinivas S (2004). Active cell migration drives the unilateral movements of the anterior visceral endoderm. Development.

[R30] Stegmaier J (2016). Real-Time Three-Dimensional Cell Segmentation in Large-Scale Microscopy Data of Developing Embryos. Dev Cell.

[R31] Strnad P (2016). Inverted light-sheet microscope for imaging mouse pre-implantation development. Nat Methods.

[R32] Tam PP (1998). Postimplantation mouse development: whole embryo culture and micro-manipulation. Int J Dev Biol.

[R33] Truong TV (2011). Deep and fast live imaging with two-photon scanned light-sheet microscopy. Nat Methods.

[R34] Udan RS (2014). Quantitative imaging of cell dynamics in mouse embryos using light-sheet microscopy. Development.

[R35] Vettenburg T (2014). Light-sheet microscopy using an Airy beam. Nat Methods.

[R36] Watanabe T (2014). Limited predictive value of blastomere angle of division in trophectoderm and inner cell mass specification. Development.

[R37] Wolff C (2018). Multi-view light-sheet imaging and tracking with the MaMuT software reveals the cell lineage of a direct developing arthropod limb. Elife.

[R38] Xenopoulos P, Nowotschin S, Hadjantonakis AK (2012). Live imaging fluorescent proteins in early mouse embryos. Methods Enzymol.

